# Investigation of an outbreak of metallo-β-lactamase producing *Pseudomonas aeruginosa* linked to the water distribution system in a Hematopoietic Stem Cell Transplantation Unit

**DOI:** 10.1017/ash.2024.378

**Published:** 2024-09-12

**Authors:** Daniela Santonato, Ivana Martinelli, Alejandra Quevedo, Roxana Sadorin, Andrea Novau, Leonardo Fabbro, María de los Ángeles Cuello Mena, Vanessa Araoz Sanchez, Wanda Cornistein

**Affiliations:** 1 Infection Control Department, Hospital Universitario Austral. Pilar, Buenos Aires, Argentina; 2 Department of Microbiology, Hospital Universitario Austral. Pilar, Buenos Aires, Argentina; 3 Department of Haematology and Hematopoietic Stem Cell Transplantation, Hospital Universitario Austral. Pilar, Buenos Aires, Argentina

## Abstract

**Introduction:**

*Pseudomonas aeruginosa* (PA) is an opportunistic pathogen. Metallo-β-lactamase producing PA (MBL-PA) poses a problematic issue given limited available treatments. In Argentina, it accounts for less than one percent of healthcare-associated infections.

**Objectives:**

To describe an outbreak of verona integron-encoded metallo-β-lactamase (VIM) *Pseudomonas aeruginosa* in a Hematopoietic Stem Cell Transplantation Unit (HSCTU), and the strategies implemented to control it.

**Materials and methods:**

Investigation of an outbreak by MBL-PA in an HSCTU in May 2023. Active case search, environmental sampling, identification and susceptibility pattern of strains, mitigation strategies. Case: patient admitted to the HSCTU with positive sample for MBL-PA after 48 hours of admission. Mitigation strategies: biweekly rectal swabbing, contact precautions, dedicated nursing staff, waterless patient care, and disinfection of bacterial reservoirs.

**Results:**

In May 2023 two cases were identified. A retrospective search determined an additional case. One (10%) of the environmental samples was positive for VIM type MBL-PA in the drain of the hand hygiene station in the nurse’s office. Strains were susceptible to colistin and fosfomycin and intermediate to aztreonam. Incidence density (ID) of colonization and infection by MBL-PA in the HSCTU were .68/1,000 patient-days (pd) and 0, respectively, in the second semester of 2022. In the first semester of 2023, ID rose to 2.93/1,000 pd for colonization and .73/1,000 pd for infection.

Mitigation strategies aimed at reducing exposure of immunocompromised hosts to water. No new cases have been identified since.

**Conclusions:**

We report an MBL-PA outbreak probably linked to the water distribution system in an HSCTU, and mitigation strategies put in place.

## Introduction

*Pseudomonas aeruginosa* (PA) is an opportunistic pathogen, frequently causing potentially severe infections in immunocompromised hosts.^1,2^ Among the characteristics that determine its prominence as a pathogen are its predilection for moist environments, mainly the distal segments of the water distribution system,^3,4^ its potential to develop biofilm, and intrinsic resistance to antimicrobials and disinfectant agents.^5^

Recently, there has been a surge of carbapenemase-producing strains, restricting the already limited therapeutic options. Hence, carbapenem-resistant PA has been included as critical-priority bacteria in the World Health Organization priority list of antibiotic-resistant bacteria.^6^ In Argentina, metallo-β-lactamase producing PA (MBL-PA) accounts for less than one percent of the isolates of healthcare-associated infections reported to the National Laboratory of [reference].^7^ Its identification is particularly worrisome given its limited therapeutic options, which determines a mortality as high as seventy percent for bloodstream infections due to MDR-PA in hematological patients.^8^

Numerous studies have reported nosocomial outbreaks of Gram-negative bacteria linked to the water distribution system.^9–15^ This premise takes particular importance in patients subjected to hematopoietic stem cell transplantation, whose immune system is severely compromised. Previous studies have reported outbreaks in this population, linked to sink drains and transmission through contaminated surfaces.^16–18^ Prompt identification and control of outbreaks in these patients is of utmost importance as it is associated with a steep morbidity burden.

This is, to our knowledge, the first Latin-American report of an outbreak of MBL-PA, and most importantly, in a Hematopoietic Stem Cell Transplantation Unit (HSCTU).

## Objectives

To describe the investigation of an outbreak by MBL-PA in an HSCTU of a tertiary care Argentinian hospital and the strategies implemented for its containment.

## Materials and methods

Description of an outbreak in concordance with the ORION recommendations for outbreak reporting.^19^

### Setting

**Figure 1.**
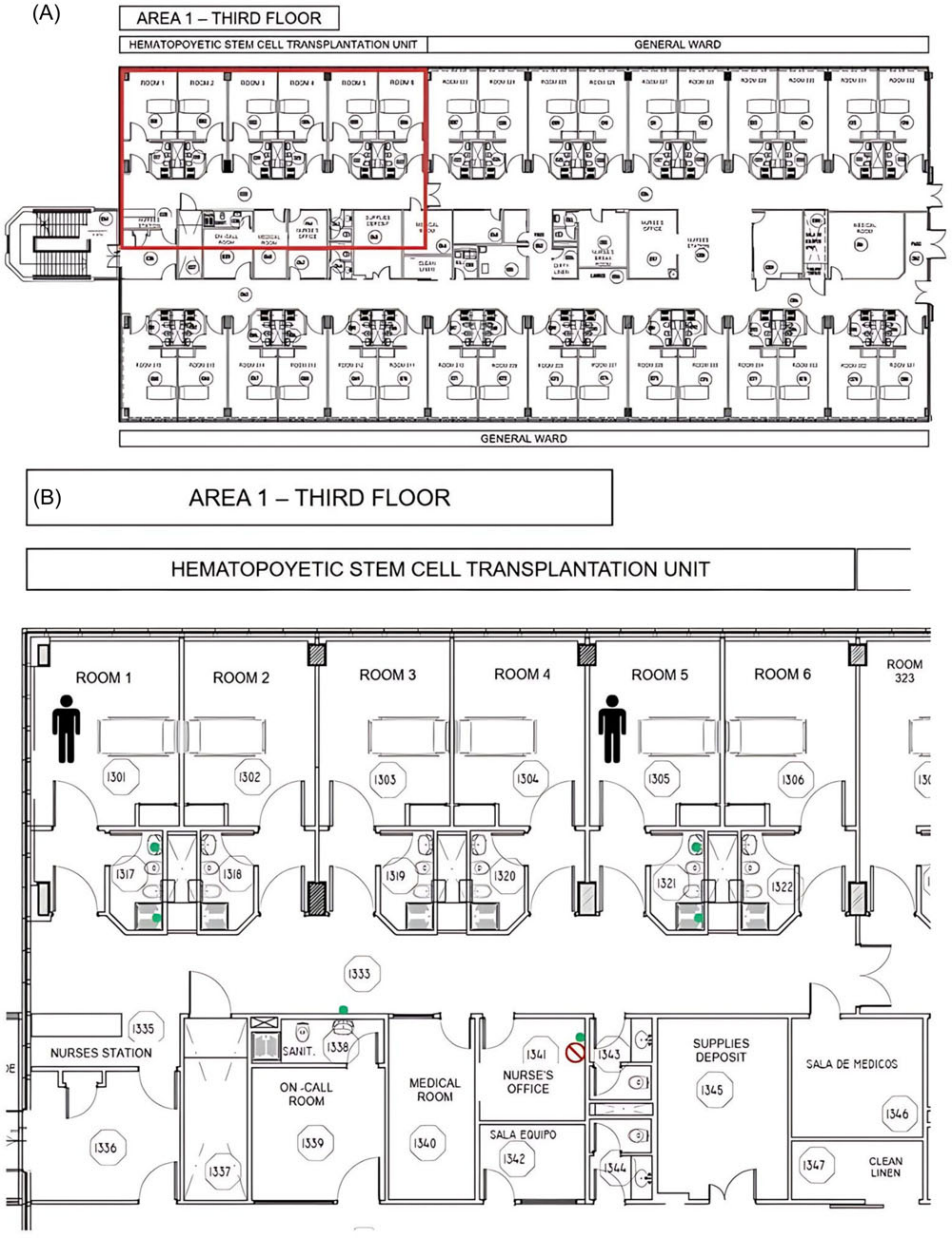
**A)** Map of the hospitalisation floor. The red box indicates the HSCTU. **B)** Map of the HSCTU. Rooms that hosted cases are marked with a human symbol. Environmental sampling points are represented with green dots. The forbidden sign marks the sink that tested positive for MBL-PA.

Hospital Universitario Austral is a 220-bed tertiary care private teaching hospital in Buenos Aires province. It has been accredited by Joint Commission International (JCI). The adult HSCTU is in a restricted area and consists of six individual rooms with an anteroom and private bathroom, a nurse’s office with a hand washing station, and an additional hand washing station in the hall (Figure 1).

Water is supplied to the institution from the provincial drinking water network and is regularly screened for the presence of bacteria as well as controlling physical and chemical characteristics. Within the institution, in accordance with JCI standards, water is tested every three months for the presence of total coliforms, PA, *Escherichia coli*, and mesophilic aerobic bacteria count.

The incidence density (ID) of colonization and infection by MBL-PA in the HSCTU were 0.68/1,000 patient-days (pd) and 0, respectively, in the second semester of 2022. In the first semester of 2022, the ID was 0 for colonization and infection.

As a standard of care, intestinal carriage screening for multidrug-resistant organisms is carried out upon admission in patients with recent hospitalization (up to ninety days before admission) in another health-care institution, as well as for patients under hemodialysis treatment. These patients are placed under contact precautions until the final lecture of the culture. Additionally, in the Intensive Care Unit and the HSCTU, patients are screened on a weekly basis.

Multidrug-resistant organisms include extended-spectrum β-lactamase producing *Enterobacterales*, vancomycin-resistant *Enterococcus* spp., carbapenemase-producing *Enterobacterales*, carbapenem-resistant PA and carbapenem-resistant *Acinetobacter baumannii*.

### Infection control measures

The implemented procedures were carried out in concordance with the recommendations by the Infection Control Department (ICD).

#### Screening

By institutional norm, patients are screened for intestinal carriage of multidrug-resistant organisms (MDRO) upon admission to the unit and weekly thereafter. Additionally, rectal swabbing was repeated on Thursdays when the Unit was hosting a patient colonized or infected by MDRO. Once the outbreak was identified, biweekly intestinal carriage screening was adopted as a norm. Screening samples are processed as described below.

#### Contact precautions

Detection of MBL-PA prompted enhanced infection control measures including contact precautions and dedicated nursing staff for cases, further restriction of access to the Unit, and limited entry to the isolation rooms. Moreover, personal protective elements were relocated to the hall from anterooms. The reasoning was that, due to a construction shortfall, bathroom doors opened to the anteroom. Hence, anterooms are potentially colonized with the patient’s microorganisms.

#### Hand hygiene compliance

In 2009 the hospital adopted the World Health Organisation Hand Hygiene Program. Accordingly, infection preventionists audit compliance with hand hygiene moments and techniques every three months. The observation modality in the HSCTU is open and direct.

#### Cleaning effectiveness

Adherence to hospital hygiene is audited through fluorescent marking and ultraviolet light.

The Table 1 shows hand hygiene compliance and cleaning effectiveness from the second trimester of 2022 to fourth trimester of 2023. The efficacy of hospital cleaning remained consistently over 80 percent. Hand hygiene compliance had diminished at the time of the outbreak and again in the third trimester of 2023. This prompted the development of scenario-based simulation training in hand hygiene.

**Table 1.** Compliance with infection prevention strategies in the Hematopoietic Stem Cell Transplantation Unit

Strategy	**3** ^**rd**^ **trimester 2022**	**4** ^**th**^ **trimester 2022**	**1** ^**st**^ **trimester 2023**	**2** ^**nd**^ **trimester 2023**	**3** ^**rd**^ **trimester 2023**	**4** ^**th**^ **trimester 2023**
Hand hygiene	86%	96%	77%	88%	70%	79%
Hospital hygiene	88%	95%	90%	98%	96%	97%

#### Education and training

Additionally, regular educational rounds were carried out by ICD personnel to reinforce compliance with hand hygiene, proper use of gloves, and donning and doffing of personal protective equipment.

### Case definition

A case was defined as a patient admitted to the HSCTU with a clinical or surveillance sample positive for MBL-PA. A case was defined as definitively nosocomial when the patient was previously negative in the same hospital stay. A case was defined as probably nosocomial when the patient had no previous negative sample during the same hospital stay and the first positive sample was retrieved more than 48 hours after patient admission. A case was defined as community acquired when the positive sample was obtained within 48 hours of admission to the unit in a patient without hospital stays in the last three months. Finally, a case was attributed to another institution when a positive sample was obtained within 48 hours of admission to the Unit in a patient hospitalized in another healthcare institution in the past three months. A case was attributed to the HSCTU when the first positive sample was collected more than 48 hours after the patient’s admission to the Unit, either during or after the stay in the HSCTU. All positive cases were followed until discharge or death.

### Environmental sampling

Once the outbreak was identified, environmental sampling was undertaken aimed at determining the reservoir of the causative agent. Given the predilection of PA to moist environments, infection preventionists identified potential reservoirs in the HSCTU to be sampled. Altogether, 10 samples were collected from faucets and sink drains of the rooms hosting cases and drains and faucets of hand washing stations in the hallway and nurse’s office.

### Bacterial identification, molecular characterization, and genomic analysis

Blood cultures were incubated using the system BD BACTEC with detection by fluorescence. Bacterial isolates were identified by matrix-assisted laser desorption ionization time-of-flight mass spectrometry (MALDI-TOF). Antimicrobial susceptibility testing was performed in accordance with European Committee on Antimicrobial Susceptibility Testing guidelines by disk susceptibility testing or Phoenix.

Screening samples were inoculated in CHROMagar™ mSuperCARBA™, a selective and differential chromogenic culture aimed at detecting carbapenem-resistant bacteria.

Environmental samples were inoculated in brain heart infusion broth and incubated at 35ºC for 24 hours. Afterward, the tubes were vortexed for thirty seconds and the tubes exhibiting turbidity were inoculated in CHROMagar™ mSuperCARBA™ medium.

Both screening and environmental samples inoculated in CHROMagar™ mSuperCARBA™ were incubated in aerobic conditions at 35°C for 48 hours. Bacterial isolates were identified by MALDI-TOF. Carbapenemase production was determined using Blue Carba Test, according to the protocol by the National Laboratory of Reference in Antimicrobials, INEI-ANLIS “Dr. Carlos G. Malbrán”.

Antimicrobial susceptibility testing was performed by disk susceptibility testing to imipenem, meropenem, boronic acid, EDTA, piperacillin/-tazobactam, and ertapenem in accordance with Kirby–Bauer method.

When necessary, NG-test ® CARBA 5, a qualitative lateral flow immunoassay, was applied for the detection and differentiation of carbapenemases families NDM, IMP, verona integron-encoded metallo-β-lactamase (VIM), OXA-48, and KPC.

### Ethical considerations

The study was classified as an outbreak investigation and, as such, was exempted from ethics committee approval.

## Results

### Outbreak description

In May 2023 two patients were identified as colonized or infected by VIM type MBL-PA in the HSCTU. Both cases were defined as definitely nosocomial and attributed to the HSCTU (Figure 1). A retrospective analysis determined the existence of three additional cases, one categorized as definitely nosocomial and two acquired in another healthcare institution. In the first semester of 2023, the ID rose to 2.93/1,000 pd for colonization and .73/1,000 pd for infection.

The clinical and demographic characteristics of the three nosocomial cases attributed to the HSCTU are included in Table 2. The mean age was 28 (range 17–41 years). Two-thirds were male. One case presented infection, and died, due to a bloodstream infection by MBL-PA, and two presented intestinal carriage.

**Table 2.** Demographics and clinical characteristics of cases acquired in the Hematopoietic Stem Cell Transplantation Unit (n = 3)

Patient	Sex	Age (years)	Date of positive culture	Type of culture	Outcome	Comorbidities
1	F	41	02/01/2023	Rectal swab	Discharge	AML - Induction
2	M	17	06/05/2023	Blood cultures	Death	T-ALL – Allo-SCT
3	M	25	08/05/2023	Rectal swab	Discharge	B-ALL – Allo-SCT

AML, acute myeloid leukemia; T-ALL, T-cell acute lymphoblastic leukemia; B-ALL, B-cell acute lymphoblastic leukemia; Allo-SCT, allogeneic hematopoietic stem cell transplantation.

### Environmental investigation

From the ten environmental samples obtained, one yielded positive for VIM type MBL-PA (10%) in the drain of the sink in the nurse’s office. The figure represents the sampling sites in the HSCTU.

The strains from patients and the environment were susceptible to colistin and fosfomycin, and intermediate to aztreonam. The three strains were sent to the National Laboratory of Reference for further analysis to confirm the outbreak. Nonetheless, at the time of writing this article, we still do not count on the results.

### Mitigation strategies

In addition to the infection control measures previously described, the identification of the bacteria in water reservoirs prompted the introduction of targeted interventions. Hence, the sinks in the hallway and nurse’s office were blocked and staff received instructions on water-free attention. In addition, the structural audit revealed that, mistakenly, diffusers had been installed in the rooms, and have since been removed to reduce the risk of aerosolization of microorganisms.

#### Disinfection of reservoirs

Another component was aimed at controlling water reservoirs. Thus, from June 2023, the HSCTU was included in the drain disinfection protocol. This protocol is carried out monthly by Infection Control professionals in critical areas and consists of pouring 500 ml of bleach down the drains of hand hygiene sinks and allowing the disinfectant to act for 30 minutes. Moreover, an institution-wide initiative was put in place to reduce the burden of MDRO in nurse’s offices. Thus, every six months, the facility maintenance and structure unit changes siphons, strains, and diffusers after mechanical cleaning and disinfection of the stations. After the first change, control environmental samples were taken and, regretfully, the drain of the nurse’s office tested positive for MBL-PA. The procedure was repeated, and controls thereafter were negative. Yet, given the potential risk for the patients, the sink remained blocked.

## Discussion

We present an outbreak by MBL-PAprobably linked to the sink drains in the HSCTU.

The role of water and, particularly, the water distribution system as a reservoir and transmission route of nosocomial outbreaks has become increasingly relevant in recent years. These outbreaks affect most frequently immunocompromised hosts and pose a significant risk of mortality. A recent study reported an outbreak by PA in a Haematology-Oncology ward linked to contaminated medication trays, which reinforces the importance of promptly detecting and controlling water reservoirs.^15^

In Argentina, MBL-PA) accounts for less than one percent of the isolates of healthcare-associated infections reported to the national laboratory of [reference].^7^

In our institution, the microorganism was probably introduced by a patient and established itself in the drains. Thereafter, likely from contaminated hands or medication trays, it was transferred to the affected patients. Once identified, the ICD put in place a series of measures aimed at controlling the outbreak. Due to an outbreak of *Fusarium* spp. infections in the Unit in 2018, several preventive interventions were already implemented. Accordingly, neutropenic patient exposure to water is restricted to showers, and this is only allowed in patients without cutaneous breaches and with the precaution of avoiding submerging the feet in water. Mineral water is provided for consumption and oral hygiene.

Before the SARS-CoV-2 pandemic, siphons and drains in nurse’s offices were routinely maintained. As a standard operative procedure, maintenance staff changed siphons, strains, and diffusers and cleaned the drains of hand hygiene stations once a year. It is not clear why this procedure was abandoned during the pandemic. A hospital-wide point prevalence study carried out after finding the positive drain swab determined a prevalence of carbapenem-resistant Gram-negative organisms in office drains of 65%. This prompted the development of a multimodal strategy aimed at controlling the burden of MDRO in the drains. The key components are:

- System change
  - Siphons, strain, and diffuser change in hand hygiene stations of nurse’s offices. Twice a year.
  - Uncoupling, mechanical cleaning, and disinfection of drains in the HSCTU rooms, every three months.
  - Monthly disinfection of drains in nurse’s offices and rooms of the SCTU and intensive care unit with bleach.
  - Creation and socialization of a list including solutions and where to discard them.
- Training and education
  - Education of staff on proper discarding of solutions
  - Information on MDRO and their predilection to drains and how to halt their development aimed at nurses, physicians, maintenance staff, and cleaning staff.
  - Hand hygiene training in the simulation rooms
- Monitoring of infection control practices and feedback
  - Periodical audit of compliance with hand hygiene, contact precautions, and cleaning efficacy.
  - Biweekly screening of intestinal carriage of MDRO.
  - Active surveillance of MDRO infections
  - Environmental sampling of drains in nurse’s offices and rooms of the SCTU.
  - Feedback of results every three months in the SCTU, as well as the Infection Control Committee and meetings with the cleaning staff.
- Workplace reminders
  - Posters indicating hand hygiene moments and technique
  - Posters in nurse’s office son appropriate discarding of solutions
- Safety climate
  - Multidisciplinary work team aligned with the primary objective
  - Consideration of the team members’ opinions and suggestions

This study is not exempted from limitations. First, at the time of submitting this article, we do not count on the genetic study of the bacterial strains. Availability of whole genome sequencing is lacking in Argentina, and the National Laboratory of Reference is, understandably, saturated. However, the temporal association and susceptibility analysis, as well as the type of MBL detected, allow us to suspect that the strains are indeed related. Second, even if we have not had new nosocomial cases since the sixteenth of May 2023, the relatively short study period of study and small sample size do not permit us to determine that the implemented measures have been successful in controlling the outbreak. Yet, since then, we have implemented a hospital-wide multimodal strategy to reduce the burden of MDRO in the drains, and have conducted several environmental samples in the unit, none of which were positive for MBL-PA. Third, the small sample size of three patients halted our intention to fully understand the epidemiology and risk factors associated with MBL-PA infections. Nonetheless, we continue to perform active surveillance of infections in the hospital, as well as surveillance of colonization in the HSCTU and Intensive Care Unit which should allow us to increment the sample size and compare different populations.

Several studies have addressed disinfection of drains.^9–15^ The authors reported chemical and physical disinfection methods, frequently in combination. Nonetheless, most mitigation strategies fail in the long term if they are not rigorously maintained. Moreover, studies in which bleach was used, differed in the concentration of the product and frequency.^14^ Thus, to date, there is not a standardized procedure for disinfecting drains with bleach. In the future, we will attempt to address this issue.

## Conclusions

We report an outbreak by Metallo- β-lactamase producing PA probably linked to the water distribution system of an HSCTU, and the mitigation strategies introduced.
